# Internet Use and Subjective Well-Being of the Elderly: An Analysis of the Mediating Effect Based on Social Capital

**DOI:** 10.3390/ijerph191912087

**Published:** 2022-09-24

**Authors:** Yang Yang, Dan Zeng, Fan Yang

**Affiliations:** Research Institute of Social Development, Southwestern University of Finance and Economics, Chengdu 611130, China

**Keywords:** Internet use, elderly, subjective well-being, social capital, mediation effect

## Abstract

At present, the Internet has a profound impact on the lifestyle and ideas of the elderly and has an important impact on the subjective well-being of the elderly. Based on the data of 2020 China Family Panel Studies (CFPS), 4463 elderly people aged 60 years and above were selected in the empirical research, including 2321 males and 2142 females. The average age was 68.07 (SD = 5.75). The ordinary least square regression (OLS) model was used to test the effect of Internet use on the subjective well-being of the elderly; the structural equation model was used to analyze the influence path of social capital in the process of Internet use affecting the subjective well-being of the elderly, and the Sobel test is used to further verify the mediating effect of social capital. The results indicated the important positive effect of Internet use on the improvement of the subjective well-being of the elderly and confirms the mediating role of social capital in the process of Internet use affecting the subjective well-being of the elderly. Among them, the mediating effects of bonding social capital and bridging social capital accounted for 18.69% and 28.17%, respectively. It can be seen that the mediating effect of bridging social capital is significantly higher than that of bonding social capital. Marital status moderates the latter half of the mediation mechanism of “Internet use–bridging social capital–subjective well-being of the elderly”, that is, “bridging social capital–subjective well-being of the elderly.”

## 1. Introduction

Since the start of the 21st century, the aging of the population in China has been increasing; at the same time, the process of digitalization and informatization in China has been accelerating. China is the world’s most populous country. At the same time, the proportion of China’s elderly population in the world’s elderly population ranks among the top in the world. In addition, China’s Internet technology is developing rapidly, and the number of netizens keeps growing. As of December 2021, the total number of mobile phone base stations in China has reached 9.96 million, the number of Internet broadband access ports has reached 1.018 billion, and the total length of optical cable lines has reached 54.88 million kilometers; in the same period, the number of netizens has reached 1.032 billion, and the Internet penetration rate has reached 73.0% [1].

The collision between the development of social digitalization and the aging structure is more prominent. According to the seventh national census in China, there are 260 million people aged 60 years or above in China, accounting for 18.7% of the country’s population. By December 2021, the number of Internet users aged 60 years and above in China reached 119 million, accounting for 11.5% of the total netizens, and the Internet penetration rate of the elderly population aged 60 and above reached 43.2%; the five most commonly used applications by elderly netizens are instant messaging, online video, Internet government services, online news, and online payment, with the utilization rates reaching 90.6%, 84.8%, 80.8%, 77.9%, and 70.6%, respectively [1]. The scale of China’s elderly Internet users continues to expand, and the Internet continues to infiltrate the lives of the elderly.

Existing studies show that Internet use contributes to the improvement of happiness of the elderly [2,3]. However, there are few studies on how Internet use affects the happiness of the elderly. This paper attempts to analyze the relationship between Internet use and the happiness of the elderly from the perspective of social capital.

There is a close relationship between Internet use, social capital, and the subjective well-being of the elderly. First, social capital has an important impact on the material and spiritual life of the elderly, thus affecting their happiness. In terms of material life, the elderly are highly dependent on social capital and need to rely on social capital to obtain economic support and life care [4]. In terms of spiritual life, social capital can effectively promote the social inclusion and social participation of the elderly, change their vulnerable status to a certain extent, and provide them with emotional comfort [5]. The improvement of both the material living standards and spiritual life of the elderly will certainly affect their subjective well-being. Therefore, it can be considered that social capital can directly or indirectly improve the subjective well-being of the elderly in both material and spiritual life.

Furthermore, Internet use is an important means to change the plight of the elderly’s lack of social capital. As the social role of the elderly changes, the breadth and quantity of their social connections also decrease, so the stock of social capital of the elderly shows a declining trend [6,7]. At the same time, the individual characteristics of the elderly group, such as the reduction of personal economic resources, deteriorating physical health, cognitive decline, and other factors, will reduce the elderly’s social participation and increase their risk of social isolation [8,9].

Internet use can increase older people’s social engagement, helping them build and maintain social relationships and increasing their social capital [10]. Specifically, this is mainly reflected in two aspects: first, Internet use helps to strengthen the relationship between the elderly and their family members, mitigating the risk of weakening the social capital of the elderly. For example, Internet use can improve communication efficiency and frequency between the elderly and family members, help strengthen the close relationship between family members [11], and enable them to obtain more financial support and emotional comfort [12]. Second, Internet use helps the elderly to expand social interaction contacts, reduce the risk of social isolation caused by age factors [13], increasing social capital. Internet use can meet the immediate social interaction needs of the elderly, help the elderly to integrate into social life, and improve social adaptability [14]. These more flexible and diverse interactive methods and interactive content also promote the social participation of the elderly and help them acquire new social roles [15]. At the same time, diverse online communities and social networks provide the elderly with rich social support possibilities [16].

Based on the above, the main goal of this study is to analyze the influence mechanism of Internet use on the subjective well-being of the elderly and try to demonstrate the mediating role of social capital in Internet use and the subjective well-being of the elderly. Therefore, this paper introduces social capital into the analysis framework to investigate the impact of Internet use on the subjective well-being of the elderly, focusing on the following questions: First, is the impact of Internet use on the subjective well-being of the elderly positive? Second, is there a mediating effect of social capital in the relationship between Internet use and the subjective well-being of the elderly? If there is a mediating effect, how does social capital play a role in the relationship between Internet use and the subjective well-being of the elderly?

## 2. Literature Review and Research Hypothesis

### 2.1. Internet Use and Subjective Well-Being

A large number of studies have found that Internet use has a positive effect on the improvement of subjective well-being [17,18,19,20]. By sorting out the previous literature, it is found that the main paths of Internet use affecting subjective well-being include four aspects.

First, Internet use will enhance the individual’s subjective well-being by promoting the acquisition and dissemination of information and strengthening interpersonal communication and connection. For example, rural residents can obtain knowledge and skills that can help improve human capital levels through the Internet and enhance individual subjective well-being [21]. For the elderly group, with the help of the Internet platform, the elderly can obtain more knowledge related to health and master more health information [22,23].

Second, Internet use affects individuals’ social perception and mentality, which in turn has an impact on individuals’ subjective well-being. In terms of social perception, Internet use improves subjective well-being through class identification, and socioeconomic status and social connection both play a partial mediating role in the relationship between Internet use and subjective well-being [24]. There is also a suggestion that Internet use may affect older adults’ life satisfaction by reducing older adults’ perceptions of social justice [25]. In addition, Internet use can also enhance the subjective well-being of older adults by enhancing social trust [26]. Besides, Internet use can help individuals reduce their focus on their material life or income, improve their sense of self-identity and satisfaction, reduce loneliness and depression, help them maintain a good mentality and improve their life satisfaction, and directly improve individuals’ subjective happiness [27,28,29].

Third, Internet use increases subjective well-being by expanding non-agricultural employment and increasing household income. For example, Internet use helps laborers find jobs through the Internet, expands private social connections to obtain more employment channels [30], reduces the cost of rural laborers to obtain various employment information [31], promotes non-agricultural employment of rural laborers on the whole [32,33], and increase family income [34].

Fourth, Internet use can enhance individuals’ social participation and interaction, and enhance social support and social connection, thus also improving individual subjective well-being [35]. For example, Internet use can facilitate intergenerational communication and exchange, thereby enhancing subjective well-being [36,37]. Especially for the elderly, Internet use can enhance their ability to exchange emotional support at a distance, maintain close family relations, expand social activities and interpersonal communication, improve subjective social adaptation, and bring positive emotional perception, thus improving subjective well-being [38,39,40].

Based on the abovementioned analysis, this paper proposes:

**Hypothesis** **1.** *Internet use can improve the subjective well-being of the elderly.*

### 2.2. Social Capital and Subjective Well-Being

From the analysis of the impact of Internet use on subjective well-being, it can be seen that no matter which path is taken, it is more or less associated with social capital. The concept of social capital was first formally put forward by Bourdieu. He believes that social capital is “a collection of actual or potential resources, which are related to the mutually familiar or recognized network of social members. It provides corresponding social support for each social member from the perspective of collective ownership and provides the evidence for social members to win reputation” [41]. Social capital differs from other capital in that it exists in agents’ social relationships, especially in social networks [42]. On the basis of this concept, many scholars have clearly defined social capital. This paper adopts Adler and Kwon’s definition and classification of social capital: “The aggregation of actual and potential resources in a specific network consisting of mutual understanding and mutual cognition” [43]. Based on this definition, social capital can be divided into getting together social capital and bridging social capital. Bonding social capital refers to the social capital generated based on strong relationships, including husband–wife relationships, family member relationships, kinship relationships, and other strong social relations, belonging to the closed network structure. Bridging social capital refers to connecting the social capital brought by the weak connections across social boundaries, that is, the loose interpersonal relationships, which belong to the open network structure [44].

The increase in social capital contributes to the improvement of individuals’ subjective well-being [45,46]. To sum up, the influence of social capital on individuals’ subjective well-being is mainly reflected in the following aspects. First, social capital can improve individual subjective well-being by improving their economic conditions [47]. Second, social capital can improve individuals’ subjective well-being by alleviating psychological pressure, loneliness, and anxiety [48]. Third, social capital can establish and enhance the connection and trust among social members and enhance the sense of belonging and social inclusion, thus promoting the improvement of individuals’ subjective well-being [46,49].

From the classification of social capital, both the bonding social capital and the bridging social capital have a positive impact on individuals’ subjective well-being, but the degree of impact is slightly different. Bonding social capital plays a positive role in alleviating the loneliness of the elderly, and individual bonding social capital is more likely to be considered an important factor in improving the subjective well-being of the elderly [50]. Bridging social capital plays a more important role in cross-ethnic and cross-cultural social communication and interpersonal interaction, which is more conducive to the social inclusion of individuals, thus improving their subjective well-being [51]. By contrast, bridging social capital may have a greater positive impact on the subjective well-being of the elderly. For example, bridging social capital can improve individuals’ economic well-being and enhance their sense of social participation and social inclusion more than converging social capital [52].

### 2.3. Internet Use and Social Capital

Internet use is an important means of expanding social capital. Social capital can only be maintained, developed, and functional in interaction. Older people who use the Internet more often have higher levels of social capital than those who use the Internet less often [53]. For the elderly, whose offline interactions gradually decrease, Internet use can enhance online interactions and give full play to the connecting and transforming function of social capital, to better maintain and expand social network relationships [54].

The impact of Internet use on social capital is reflected in two aspects: maintaining strong relationships and establishing and expanding weak relationships. In terms of bonding social capital, Internet use can strengthen strong relationships, maintain connections with relatives and friends who live more dispersedly, provide emotional and financial support, and enhance bonding social capital [55]. Online connections through Internet use, for example, form tighter networks of strong relationships than theoretically predicted [56].

In terms of bridging social capital, Internet use can establish and maintain weak ties, promote information exchange with members of weak ties, broaden access to resources and information, expand social networks, and enhance bridging social capital, thus improving the heterogeneity, extensiveness, and accessibility of individual social capital [57,58,59]. At the same time, Internet use also provides a new platform for social interaction and participation, promoting online and offline social activities, enhancing social connections, and enhancing bridging social capital [60].

Based on the abovementioned analysis, this paper proposes the following hypotheses:

**Hypothesis** **2.** *Internet use enhances the social capital of the elderly, thus improving their subjective well-being.*

**Hypothesis** **2A.** *Internet use enhances the bonding social capital of the elderly, thus enhancing their subjective well-being.*

**Hypothesis** **2B.** *Internet use enhances the bridging social capital of the elderly, thus enhancing their subjective well-being.*

In addition, marital status affects the relationship between individual social capital status and individuals’ subjective well-being. Good marital status is helpful for improving individual social capital and enhancing the effect of social capital on subjective well-being [61]. The reason is that marriage plays a very important role in integrating the social resources of the couple [62]. An individual’s social network expands when they join their spouse’s social network [63], and couples can share the local resources and networks of their spouses and their families [64]. In particular, inter-regional marriage increases the local social interaction and social participation of the floating population, builds a local relationship network, accumulates social capital, and strengthens the positive impact of social capital on subjective well-being [65]. Thus, marriage among older adults may strengthen the relationship between social capital and subjective well-being. Compared with the elderly without a spouse, the subjective well-being of the elderly with a spouse is more significantly affected by the positive effect of bonding social capital and bridging social capital.

Based on the abovementioned analysis, this paper proposes:

**Hypothesis** **3.** *Marital status plays a moderating role in the latter half of the mediation pathway (bridging social capital–subjective well-being).*

### 2.4. Internet Use, Social Capital, and Subjective Well-Being

**Figure 1:**
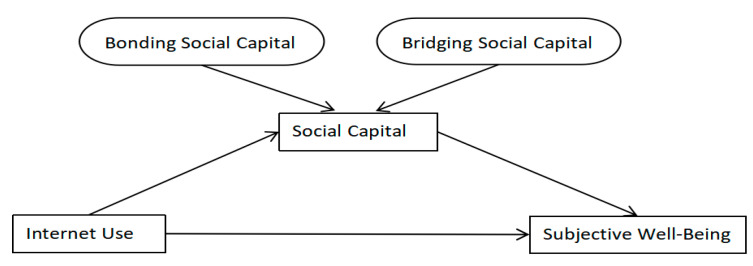
Theoretical schematic diagram of the core variable.

There is a close connection between Internet use, social capital, and subjective well-being. Internet use not only affects the social capital of the elderly but also affects the subjective well-being of the elderly. At the same time, the Internet is a convenient and effective means of maintaining existing social relationships or establishing new ones and is conducive to the accumulation of social capital [66]. The elderly can expand their social network through social capital, realize social interaction, and obtain emotional support and reciprocity of resources, thereby enhancing their subjective well-being. It is believed that Internet use can enhance the subjective well-being of older adults by improving social capital. First, Internet use can enhance the subjective well-being of the elderly by increasing the frequency of contact between the elderly and strong ties, strengthening emotional comfort and economic support, that is, maintaining and consolidating bonding social capital. Second, Internet use can enhance the subjective well-being of the elderly by enhancing the connection between the elderly and weak tie groups, enhancing social participation and social interaction, that is, expanding and building bridging social capital (see Figure 1).

## 3. Research Design

### 3.1. Data Sources

This paper uses the questionnaire data from China Family Panel Studies (CFPS) in 2020. CFPS is a national and large-scale social tracking survey project implemented by the Chinese Center for Social Sciences Survey (ISSS) of Peking University, covering 31 provinces/municipalities/autonomous regions. The CFPS project follows relevant regulations, regularly submits applications for ethical review or continuous review to the Institutional Review Board of Peking University (PU IRB) and carries out corresponding data collection upon approval of the ethical review [67]. Considering the great regional differences in Chinese society, and in order to reduce the operating cost of the survey, CFPS sampling adopts the probability sampling method of implicit stratification, multi-stage, multi-level, and proportional to population size (PPS). Administrative division and socio-economic level are the main stratified variables. Each sub-sample of this survey was taken through three stages. The first stage sample (PSU) is the administrative district or county, the second stage sample (SSU) is the administrative village or neighborhood committee, and the third stage (end) sample (TSU) is family households. In 2020, the sample size of the adult questionnaire was 28,590, among which the sample size of the elderly was 6976. As the research object is the elderly, the sample data are screened in this paper, and people over 60 years are retained. After removing missing values and outliers of key variables, a total of 4463 samples are obtained.

### 3.2. Measurement of Variables

#### 3.2.1. Core explanatory Variable: Internet Use

Internet use means whether you surf the Internet with a web device. Referring to previous research, the core independent variable of this paper “Internet use” is measured by “yes or no” [68,69]. The questions about personal Internet use in the questionnaire are as follows: “Do you use mobile devices to surf the Internet”? “Do you use a computer to access the Internet”? As long as one of these two behaviors is confirmed, it is identified as having online behavior. Internet usage is set to a binary dummy variable, with online behavior assigned 1 and 0 assigned otherwise.

#### 3.2.2. Explained Variables: Subjective Well-Being

The subjective well-being of the elderly was measured by the question “How happy do you think you are?”. Respondents indicated their answers on a scale of 0–10. The higher the score, the stronger the sense of happiness. In most cases, there is a high similarity and consistency between the respondents’ responses and the experts’ assessment results of the respondents [70].

#### 3.2.3. Mediating Variable: Social Capital

Bonding social capital mainly exists in groups with close emotional relationships, such as family members and close friends, and has stronger homogeneity. Convergent social capital emphasizes the internal connections among members of strong relationships and focuses on emotional support among members [71]. Therefore, bonding social capital is generally measured by the relationship with relatives and friends, and the boundary of social interaction is defined as family or relatives [72,73,74]. Due to the limitations of the questionnaire, we selected the frequency of contact with each child to measure bonding social capital. “How often have you contacted Child Name by phone, SMS, letter, or email in the past six months?” The options were almost daily, 3–4 times a week, 1–2 times a week, 2–3 times a month, once a month, once every few months, and never. We asked about the frequency of contact with each child in turn. The options were coded from 1 to 7. The higher the frequency of contact between parents and children, the higher the assigned value. Then the frequency of contact between the elderly and their individual children was added up and divided by the number of children to obtain the frequency of contact with their children. To avoid the influence of outliers on the regression results, a logarithm of the mean contact frequency between the elderly and their children was taken. The higher the value, the higher the frequency of contact between the elderly and their children, and the greater the bonding social capital.

Bridging social capital focuses more on the loose and weak connections between individuals. Compared with bonding social capital, bridging social capital is more heterogeneous and can provide individuals with useful information and resources [75]. Popularity reflects whether an individual fits in with others. Individuals with rich interpersonal relationships have a wider range of social relationships and are more able to mobilize social resources and obtain information. The broader the individual social relations, the richer the social capital bridged [76]. Generally speaking, an individual’s good “popularity” in a group or social circle is an important form of capital for an individual to obtain more resources [77]. This paper uses kinship to measure the status of bridging social capital in the elderly. In the questionnaire, the question “How popular do you think you are?” was asked, and the respondents provided a score of 0–10. The higher the score, the richer the bridging social capital of the elderly.

#### 3.2.4. Control Variables

Gender, age, marital status, urban and rural attributes, education level, and other basic individual characteristics were included in the control variables. Meanwhile, having a healthy physical and mental state is an important factor for the elderly to improve their happiness [78]. Their economic situation will also affect the subjective well-being of the elderly. Relative income status and subjective social are both positively correlated with happiness [79,80]. Therefore, self-rated health, relative income status, and self-rated social status were also used as control variables.

In terms of gender, female was assigned 0, and male was assigned 1. In terms of age, the elderly aged 60–69 years were classified into the younger group, with a value of 1. The elderly aged 70–79 years were classified into the middle-age group, and the value was 2. The elderly aged 80–100 years were classified into the advanced age group, and the value was 3. In terms of the urban and rural attributes of residence, the elderly living in rural areas were assigned 0, and those living in towns were assigned 1. In terms of education level, the illiterate or semi-illiterate were assigned 1, those with elementary school education were assigned 2, junior high school 3, high school 4, and college or higher 5. In terms of marital status, unmarried, divorced, and widowed individuals were classified as unaccompanied by spouse, assigned 0. Cohabitation and having a spouse were classified as accompanied by a spouse and assigned a value of 1. For the relevant question in the questionnaire, “What is your personal income level in the local area?” the respondents’ answers ranged from 1 to 5, indicating “very low” to “very high”, respectively. The higher the value, the higher the relative income level of the respondents. Self-rated social status and self-rated health were continuous variables of 1–10.

Table 1 shows the basic information of relevant variables. In terms of gender, the female elderly accounted for 47.99%, and the male accounted for 52.01%. In terms of age group, the younger group accounted for 64.60%, the middle group accounted for 30.92%, and the advanced group accounted for 4.48%. The elderly living in rural areas accounted for 50.01%, and those living in urban areas accounted for 49.99%. In terms of education level, 39.59% of the elderly were illiterate or semi-illiterate, while 22.45% had completed primary school, 21.89% junior high school, 13.11% senior high school, and 2.96% junior college or above. In terms of marital status, the unaccompanied elderly accounted for 15.19%, while those accompanied by a spouse accounted for 84.81%.

Among the mediating variables, bonding social capital ranged from 1 to 7, with a minimum value of 1, a maximum value of 7, a mean value of 4.48, and a standard deviation of 1.76. After logarithmic processing, its minimum value and maximum value were 0 and 1.95, respectively, with a mean value of 1.38 and a standard deviation of 0.55. The minimum value and maximum value of bridging social capital were 0 and 10, respectively, the mean value was 7.41, and the standard deviation was 2.03.

**Figure 2:**
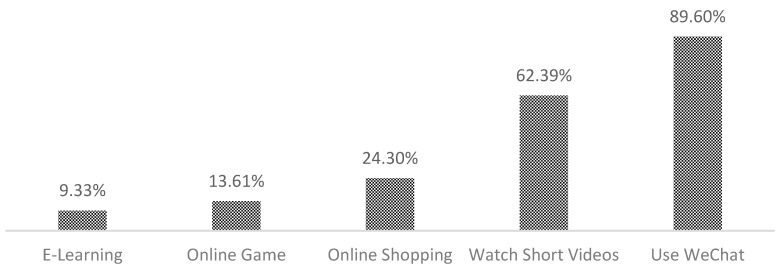
Types of Internet use among the elderly.

From the descriptive statistics, 23.08% of the elderly used the Internet, and 76.92% of the elderly did not use the Internet. As can be seen from Figure 2, among the elderly who used the Internet, 9.33% used the Internet to study, 13.61% played online games, 24.30% shopped online, 62.39% watched short videos, and 89.60% used WeChat. In other words, a small number of the elderly studied and played games on the Internet, some used the Internet to shop and watch videos, and the vast majority used the Internet to contact and communicate with others.

**Figure 3:**
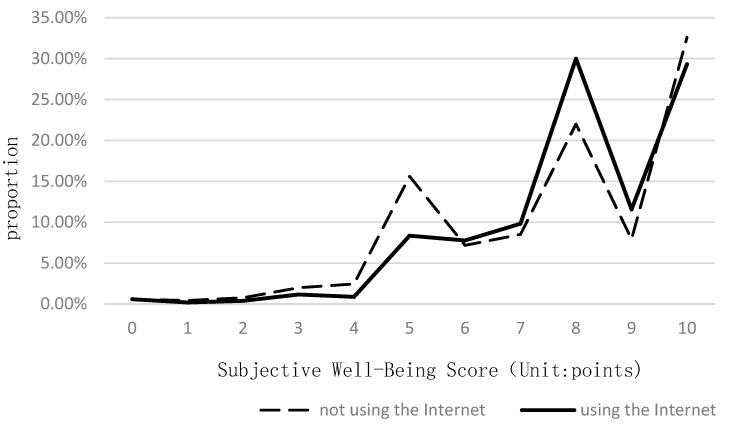
Internet use and subjective well-being scores of the elderly.

As can be seen from Figure 3, there was a certain difference in subjective well-being between the elderly who used the Internet and those who did not. Most elderly people who did not use the Internet had lower subjective well-being scores. In the elderly group with a 0–5 subjective well-being score, the proportion of the elderly who used the Internet was generally lower than that of the elderly who did not use the Internet. For example, the proportion of the elderly who did not use the Internet was 0.24, 0.37, 0.82, 1.57, and 7.26 percentage points higher than that of the elderly who used the Internet in the subjective well-being groups of 1, 2, 3, 4, and 5 points, respectively. The proportion of the elderly who used the Internet was significantly higher in the aged group with a subjective well-being score of 5–10. For example, the proportion of the elderly who used the Internet was 0.60, 1.30, 8.01, and 3.63 percentage points higher than that of the elderly who did not use the Internet in the subjective well-being groups of 6, 7, 8, and 9 points, respectively. In the elderly group with a subjective well-being score of 10, the proportion of the elderly who used the Internet was slightly lower than the proportion of the elderly who did not use the Internet by 3.28 percentage points. Overall, older adults who used the Internet had higher subjective well-being scores than those who did not use the Internet.

### 3.3. Research Methods

This study mainly investigated the influencing mechanism of Internet use on the subjective well-being of the elderly. In the first step, based on the research questions and hypotheses, ordinary least squares regression (OLS) was used to build the model. The second step is to consider the endogeneity of cross-sectional data. There is a two-way causal relationship between Internet use and subjective well-being of the elderly and between social capital and subjective well-being of the elderly. The lag term model of explained variables was used to test the possible endogeneity problems. Third, a structural equation model was used to explore the influence path and mechanism of Internet use on the subjective well-being of the elderly. Then, the Sobel test was used to examine the mediating effect of social capital. Finally, on the basis of the OLS regression model, a moderated mediating effect model was adopted, that is, the interaction terms of using or not using the Internet and marital status, as well as social capital index and marital status, were added to the OLS model to test the moderating role of marital status in the process of Internet use affecting the subjective well-being of the elderly.

## 4. Empirical Results Analysis

### 4.1. The Direct Impact of Internet Use on the Subjective Well-Being of the Elderly

OLS regression results showed that Internet use had a significant positive impact on the subjective well-being of the elderly (Table 2). According to Model 1, without controlling any variables, the regression coefficient of Internet use on subjective well-being was 0.307 (*p* < 0.01). In Model 2, control variables related to personal and social characteristics were added on the basis of Model 1. Internet use still had a significant positive impact on the subjective well-being of the elderly (*p* < 0.01).

Before the introduction of the mediation variable, the independence test of the mediation variable and the control variable was carried out, and its variance inflation factor (VIF) value was between 1.07 and 1.53. It could be considered that there was no serious multicollinearity between the mediation variable and the control variable. On the basis of Model 2, Model 3 included the mediating variable bonding social capital. At the level of 0.01, there was still a significant positive correlation between Internet use and the subjective well-being of the elderly, with a regression coefficient of 0.266. Model 4 incorporated bridging social capital on the basis of Model 2. The regression coefficient of Internet use to subjective well-being (SWB) was 0.235 (*p* < 0.01). It can be seen that there was also a significant positive correlation between Internet use and the subjective well-being of the elderly. On the basis of Model 2, Model 5 introduced both bonding social capital and bridging social capital. The results showed that the regression coefficient of Internet use on the subjective well-being of the elderly was 0.194 and significant at the level of 1%; thus, Hypothesis 1 was verified.

It is worth noting that, compared with Model 2, the regression coefficients of Internet use on subjective well-being in Model 3, Model 4, and Model 5 decreased by 0.061, 0.092, and 0.133 units, respectively. To some extent, this indicates that social capital may play a mediating role in the process of Internet use affecting the subjective well-being of the elderly, which has been preliminarily verified. The specific mechanism will be analyzed through the path analysis of the structural equation model and Sobel test to investigate the mediating effects of bonding social capital and bridging social capital.

### 4.2. Endogeneity Test

As the regression model in this study adopts cross-sectional data, endogenous problems inevitably exist. There may be mutual causality between Internet use and individuals’ subjective well-being and between social capital and subjective well-being. In other words, the Internet use can affect the subjective well-being of the elderly, and the subjective well-being of the elderly also determines their Internet use behavior to a certain extent. Social capital will affect the subjective well-being of the elderly, and the level of subjective well-being also shapes the bonding social capital and bridging social capital of the elderly to a certain extent. To solve this problem, the method of explaining variable lags behind is adopted. Introducing lag terms of explained variables into the model is one of the methods to break the bidirectional causality between the explained variables and the explained variables, and it can be assumed that the behavior of individuals at *t* + 1 is only affected by the group behavior at the t time point [81].

The observed value of Internet use in 2018 was taken as the explanatory variable, and the observed value of bonding social capital (frequency of contacting their children) and bridging social capital (interpersonal relationships) of the elderly in 2018 was taken as the intermediary variable. The dependent variable was still the subjective well-being of the elderly in 2020. To some extent, this could avoid the influence of subjective well-being on Internet use and the influence of subjective well-being on the social capital of the elderly on the estimation results. Table 3 shows the lag term model for testing endogeneity problems. Model 6 introduced Internet use in 2018 as an explanatory variable, and the results showed that the subjective well-being of the elderly who used the Internet was significantly increased by 0.223 points compared with that of the elderly who did not use the Internet (*p* < 0.05). Based on Model 6, Model 7 introduced the bonding social capital and bridging social capital of the elderly in 2018. The regression coefficient of the impact of Internet use on the subjective well-being of the elderly was 0.188 and significant at the level of 10%. The regression coefficients of bonding social capital and bridging social capital on the subjective well-being of the elderly were 0.193 and 0.167, respectively, and both were significant at the level of 1%.

In conclusion, the results of endogeneity analysis are consistent with those of models 1–5, which solves the endogeneity problem to some extent—that is, Internet use significantly improves the subjective well-being of the elderly. After introducing the social capital variable, the regression coefficient of Internet use is significantly positive and has a significant decline, and social capital may play a mediating role.

### 4.3. Indirect Effects of Internet Use on Subjective Well-Being of the Elderly

According to the abovementioned analysis, Internet use may affect the subjective well-being of the elderly through the factor of social capital. A structural equation model was used to investigate the influence of Internet use and social capital on the subjective well-being of the elderly. Then, a Sobel mediating effect test was conducted to clarify the mechanism of social capital between Internet use and the subjective well-being of the elderly. Table 4 shows the test results of the mediating effect of Internet use on the subjective well-being of the elderly.

**Figure 4:**
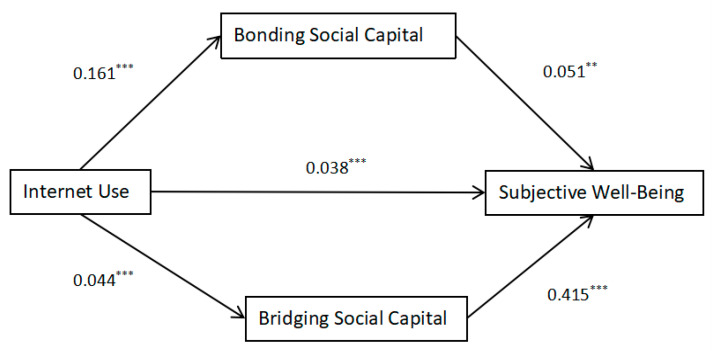
Path analysis of the influence of Internet use on the subjective well-being of the elderly. Note: *** *p* < 0.01, ** *p* < 0.05. The coefficients in the abovementioned model are standardized path coefficients.

The results show that the overall fitting of the model is good (Figure 4). The fitting index value of this model is RMSEA = 0.056; SRMR = 0.007; CFI = 0.996. The structural equation model can be adapted to the actual data. In terms of specific path coefficients, the significance levels of path coefficients of independent variables, dependent variables, and intermediate variables all passed the z test of 0.05, and the path coefficients of some control variables were not significant, but they were not deleted in consideration of the overall fitting effect of the model.

The results show that the influence of Internet use on the subjective well-being of the elderly includes direct effects and indirect effects. The standardized path coefficient of the direct influence of Internet use on the subjective well-being of the elderly was 0.038 (*p* < 0.01), indicating that Internet use enables the elderly to obtain higher subjective well-being. The indirect effect is mainly realized through the influence of social capital on the subjective well-being of the elderly. The direct effects of Internet use and social capital on the subjective well-being of the elderly are significant at the 1% level.

#### 4.3.1. The Mediating Effect of Bonding Social Capital on the Internet Use and Subjective Well-Being of the Elderly

Bonding social capital plays a significant mediating role in the elderly’s Internet use and subjective well-being. The standardized path coefficient of the direct impact of Internet use on the bonding social capital of the elderly is 0.161 (*p* < 0.01), indicating that Internet use can increase the bonding social capital of the elderly. The bonding social capital of the elderly also has a positive direct effect on their subjective well-being, and the standardized path coefficient is 0.051 (*p* < 0.01)—that is, the higher the bonding social capital of the elderly, the more likely they are to have higher subjective well-being. Therefore, Internet use can indirectly influence the subjective well-being of the elderly through bonding social capital.

Table 4 tests the Sobel mediating effect of bonding social capital. Sobel test results show that the indirect effect of Internet use through bonding social capital is significantly positive (*p* < 0.01), and the mediating effect is obvious. The indirect effect accounts for 18.69% of the total effect—that is, 18.69% of the impact of Internet use on the subjective well-being of the elderly is achieved through bonding social capital. Thus, bonding social capital plays a part in the intermediary role.

#### 4.3.2. The Mediating Effect of Bridging Social Capital on the Internet Use and Subjective Well-Being of the Elderly

Bridging social capital plays a significant mediating role in the relationship between Internet use and the subjective well-being of the elderly. The standardized coefficient of the direct impact of Internet use on the bridging social capital of the elderly is 0.044 (*p* < 0.01), indicating that Internet use can significantly improve the bridging social capital of the elderly. The bridging social capital of the elderly also has a positive direct effect on their subjective well-being, and the standardized path coefficient is 0.415 (*p* < 0.01)—that is, the higher the bridging social capital of the elderly, the more likely they are to have higher subjective well-being.

As can be seen from Table 4, Internet use has an indirect impact on the subjective well-being of the elderly through bridging social capital. The mediating effect passed the Sobel test and was significant (*p* < 0.01), and its indirect effect accounted for 28.17%, indicating that 28.17% of the influence of Internet use on the subjective well-being of the elderly was realized through the influence of bridging social capital, with bridging social capital playing a part of the mediating role. In addition, the indirect effect of bridging social capital (28.17%) was 9.48 percentage points higher than that of converging social capital (18.69%). Therefore, bridging social capital has a higher explanatory effect on the well-being effect of the elderly. This indicates that Internet use can expand the bridging social capital of the elderly, enabling them to obtain more opportunities for social activities, improve their social skills, enhance weak connections, and further strengthen social participation, thus improving their subjective well-being.

In conclusion, bonding social capital and bridging social capital play an intermediary role in the relationship between Internet use and the subjective well-being of the elderly, which can well explain how Internet use improves the subjective well-being of the elderly. Thus, Hypothesis 2 is confirmed.

### 4.4. The Moderating Effect of Marital Status

This paper introduces marital status as a moderating variable of the mediating model of “Internet use–social capital–subjective well-being”. The results of Models 6 and 7 in Table 5 show that marital status has no significant impact on the first half of the mediation pathway (Internet use–converging social capital, Internet use–bridging social capital). Therefore, it is further analyzed whether the influence path of marital status on the latter half of the intermediary mechanism (bonding social capital–subjective well-being, bridging social capital–subjective well-being) has a moderating effect.

**Figure 5:**
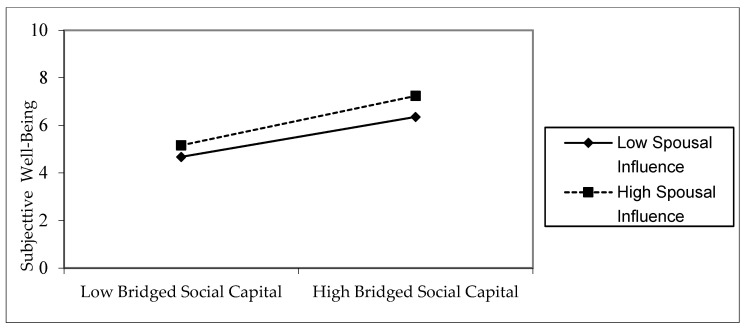
Marital status adjustment of the influence of bridging social capital on the subjective well-being of the elderly.

The regression results of Model 8 show that the interaction term between bonding social capital and marital status is not significant and does not have a moderating effect—that is, marital status does not affect the relationship between bonding social capital and the subjective well-being of the elderly. Meanwhile, according to the results of Model 9, the regression coefficient of the main effect of bridging social capital is significantly positive (*p* < 0.01), and the interaction term between bridging social capital and marital status is significantly positive (*p* < 0.05)—that is, marital status reinforces or promotes the relationship between bridging social capital and subjective well-being of the elderly, with a significant positive adjustment effect. Figure 5 further reflects the moderating effect of marital status. Compared with the unaccompanied elderly, the elderly accompanied by their spouse are more likely to experience an enhanced positive impact of bridging social capital on their subjective well-being. Therefore, the moderated mediation model is established, and marital status moderates the latter half path of the mediation model “Internet use–bridging social capital–subjective well-being”. Thus, Hypothesis 3 is partially verified.

## 5. Discussion

### 5.1. Mediating Role of Social Capital

Based on the data of the China Family Panel Studies in 2020 and from the perspective of social capital, this paper empirically analyzed the relationship between Internet use and subjective well-being of the elderly over 60 years and its influence path using a structural equation model, Sobel test, and other methods. This study found, compared with the elderly who do not use the Internet, the elderly who use the Internet have a higher degree of subjective well-being [21,82]. Internet use can significantly consolidate bonding social capital and build and expand bridging social capital, thus enhancing the subjective well-being of the elderly [83]. We believe that social capital plays a mediating role in the process of Internet use affecting the subjective well-being of the elderly for the following reasons. 

The mediating effect of bonding social capital can be seen as follows: First, Internet use provides diversified communication channels and methods for the elderly, increases the frequency and intimacy of strong relationships with relatives and friends, and enables them to obtain economic support and emotional comfort brought by strong relationships, thus improving their quality of life [84,85]. Second, Internet use provides opportunities for the elderly to enhance their bonding social capital and meet their needs for belonging and love, thus improving their subjective well-being. The Internet contains diversified information content, creates and increases topic content between the elderly and strong relationship groups, and adds common language, which helps maintain intergenerational relations and strengthens the bonding social capital [36].

The mediating role of bridging social capital can be seen as follows: First, Internet use provides a platform for social participation, expands bridging social capital, helps the elderly to carry out and participate in online group activities and offline activities derived from online group activities, and expands bridging social capital. This enriches their daily life and helps them form a more positive and optimistic attitude toward life. Second, Internet use can promote the re-socialization of the elderly. The elderly can acquire knowledge to improve their self-value from the Internet, to actively strengthen their awareness and action of social participation, strengthen their bridging social capital, and improve their subjective well-being [86]. Third, the elderly can use the Internet to establish and maintain bridging social capital across time and space, helping them find groups with common interests and values and enhancing communication and exchange with weak-ties groups. Therefore, it can alleviate social isolation and loneliness, enhance the sense of social participation and integration of the elderly, and promote the improvement of their subjective well-being [87,88]. For example, in recent years, the phenomenon of silver Internet celebrities has boomed. The elderly use Internet video social software to express themselves and present themselves and extend their social roles and relationships through interaction with their followers [89].

In addition, compared with bonding social capital, bridging social capital plays a more obvious and important role in helping the elderly to enrich their social roles, build new social relationships, strengthen social participation, and thus establish and enhance their sense of self-identity and self-worth. At the same time, Internet use is more powerful in helping the elderly to establish and stabilize social relationships and social interaction activities and can enhance bridging social capital. Therefore, the mediating effect of bridging social capital is more obvious.

From the moderating effect of marriage, it can be seen that marital status moderates the relationship between bridging social capital and the subjective well-being of the elderly. Strong family relationships are strongly associated with emotional well-being and physical health in older people [90]. For the elderly accompanied by their spouse, marriage is the link, and the couple can often share the social resources contained in their kinship relationships, friend relationships, and colleague relationships [91]. Spouses can integrate the social network resources of both parties, jointly construct and share bridging social capital, strengthen participation in social activities, and extend their weak relationship network, thus enhancing the positive impact of bridging social capital on the subjective well-being of the elderly.

Of course, Internet use can also bring some negative effects. Excessive Internet use may be detrimental to happiness [92]. First, the Internet may weaken family relations or promote the psychology of keeping up with the Joneses, thus reducing life satisfaction and subjective happiness [93]. Second, prolonged Internet use may reduce the amount of time individuals invest in face-to-face interactions, negatively impact social capital, and in severe cases lead to greater isolation and social exclusion, which in turn reduces happiness [94].

### 5.2. Relevant Policy Recommendations

It is necessary to further promote the appropriate aging of Internet use and strengthen the role of Internet use in improving subjective well-being by enhancing social capital. There are some recommends: First, it is necessary to further enhance the popularity of Internet technology among the elderly, relying on families and communities to help the elderly truly integrate into the Internet age and empower the elderly. Second, improve the initiative of the elderly to use the Internet, pay attention to the Internet needs of the elderly group, and continuously promote the aging-appropriate development of Internet equipment and functions. Third, it is necessary to give full play to the function of the Internet to expand social capital, cultivate and enhance the bonding social capital and expand the bridging social capital of the elderly, and try to avoid the establishment of bad relationships. Further, it is important to appropriately guide the elderly to use the Internet to enhance social participation, fully realize the effective acquisition of information, exchange of resources, participate in social activities, and establish social relationships in the Internet age, so as to form an active and healthy lifestyle, thereby improving the subjective well-being of the elderly.

### 5.3. Marginal Contribution of Research

The marginal contributions of this study are mainly as follows. First, this study further refines the mediating role of social capital between Internet use and the subjective well-being of the elderly. For the classification of social capital, the classification methods of cognitive social capital and structural social capital are generally adopted in academic circles. However, the proportion of related research based on the classification methods of bonding social capital and bridging social capital is relatively small. Starting from the two dimensions of bonding social capital and bridging social capital, it provides new Chinese empirical evidence for a deeper understanding of the relationship between Internet use and elderly well-being. Second, there is an urgent need to strengthen theoretical matching and empirical research on the impact of Internet use on the subjective well-being of the elderly. This study tries to overcome the possible endogeneity problem by adopting the lagged term of the explained variable and providing robust empirical evidence for understanding the relationship between the Internet, social capital, and subjective well-being.

### 5.4. Research Limitations and Research Prospects

This study, while striving to remain rigorous, suffers from several limitations. First, the explained variable lag model is used to try to solve the problem of bidirectional causality between core variables, but there are still shortcomings. For example, there may also be endogeneity issues such as sample selection bias. Second, although this study refers to the existing literature to screen control variables, there may still be some neglected related factors. Third, the measurement of social capital is limited by questionnaire data. The measurement of the two types of social capital was combined with the actual situation in China. On the one hand, with the availability of relevant data in the CFPS database, we can only select limited indicators to measure bonding social capital and bridging social capital. On the other hand, these two types of measurement indicators are obtained in the questionnaire by the respondents’ self-assessment, and there may be a gap between the self-answered questionnaire and the facts.

These below are worthy of further study. Future research can explore how the specific content of a certain Internet use affects the subjective well-being of the elderly through social capital. Of course, there may be other ways that Internet use affects the subjective well-being of the elderly, and future research may need to be further expanded and deepened. At the same time, it is better to use panel data in future related research, and the measurement of social capital should be refined according to the actual situation.

## 6. Conclusions

This study provides empirical evidence for exploring the mediating role of social capital in the process of Internet use affecting the subjective well-being of the elderly. The main conclusions are as follows. First, Internet use has a positive effect on the subjective well-being of the elderly. Second, social capital plays a significant mediating role in the process of Internet use affecting the subjective well-being of the elderly. Third, bridging social capital has a higher mediating effect than bonding social capital. It can be seen that Internet use has a more obvious effect on enhancing the subjective well-being of the elderly by exploring the depth and breadth of bridging social capital. Fourth, marital status moderates the latter half of the influence pathways of Internet use, bridging social capital and elderly subjective well-being. Marital status strengthens the influence of bridging social capital on the subjective well-being of the elderly. The positive impact of bridging social capital on subjective well-being is more obvious for the elderly accompanied by their spouse.

These research findings provide a certain reference for further promoting the elderly to enhance their social capital through Internet use to improve their subjective well-being, and to expand future related research.

## Figures and Tables

**Table 1:** Descriptive statistics of relevant variables.

Category	Variables	Variable Definition and Assignment	Mean	SD
Explanatory variables	Use the Internet	Yes = 1; no = 0	0.23	0.42
Explained variable	Subjective well-being	0–10, continuous variable	7.79	2.13
Intervening variable	Bonding social capital	Frequency of contact with children: 0–5, continuous variable	4.48	1.75
	Bridging social capital	Popularity: 0–10, continuous variable	7.41	2.03
Control variables	Gender	Male = 1; female = 0	0.52	0.50
	Age group	60–69 = 1; 70–79 = 2; more than 79 = 3	1.40	0.57
	Place of residence	Urban and rural property of residence: urban = 1; rural = 0	0.50	0.50
	The degree of education	Illiterate or semi-illiterate = 1; primary school = 2, junior high school = 3; senior high school = 4; junior college and above = 5	2.17	1.17
	Marital status	Unaccompanied by spouse (including unmarried, divorced, widowed) = 0; accompanied by spouse (including cohabitation and spouse in marriage) = 1	0.85	0.36
	Relative income level	1–5, continuous variable; the higher the value, the higher the relative income status	3.20	1.15
	Self-assessment of social status	1–5, continuous variable; the higher the value, the higher the self-rated social status	3.49	1.10
	Self-reported health	1–5, continuous variable; the higher the value, the better the self-rated health status	2.65	1.25

**Table 2:** OLS regression model of the influence of Internet use on the subjective well-being of the elderly.

Variables	Model 1	Model 2	Model 3	Model 4	Model 5
Using or not using the Internet	0.307 *** (4.06)	0.327 *** (4.08)	0.266 *** (3.29)	0.235 *** (3.24)	0.194 *** (2.65)
Gender		−0.102 (−1.60)	−0.081 (−1.28)	0.000 (0.00)	0.013 (0.23)
Age group		0.290 *** (5.31)	0.321 *** (5.87)	0.246 *** (4.99)	0.267 *** (5.40)
Education level		0.053 * (1.77)	0.040 (1.34)	0.026 (0.95)	0.017 (0.64)
Marital status		0.364 *** (4.18)	0.349 *** (4.03)	0.350 *** (4.46)	0.341 *** (4.34)
Urban or rural area		0.164 *** (2.61)	0.166 *** (2.65)	0.182 *** (3.20)	0.183 *** (3.23)
Relative income level		0.203 *** (6.42)	0.199 *** (6.30)	0.187 *** (6.52)	0.184 *** (6.43)
Self-assessment of social status		0.360 *** (10.91)	0.355 *** (10.78)	0.185 *** (6.11)	0.183 *** (6.05)
Self-reported health		0.271 *** (11.06)	0.271 *** (11.07)	0.203 *** (9.14)	0.203 *** (9.16)
Convergent social capital: Frequency of contact with children			0.289 *** (5.18)		0.197 *** (3.88)
Bridging social capital: kinship				0.437 *** (31.74)	0.434 *** (31.51)
Constant	7.715 *** (212.66)	4.228 *** (25.12)	3.860 *** (21.17)	1.919 *** (11.39)	1.685 *** (9.43)
*n*	4463	4463	4463	4463	4463
*R*-squared	0.004	0.125	0.130	0.286	0.289
*F*	16.48	70.71	66.69	178.74	164.37

Note: *** *p* < 0.01, * *p* < 0.1. The values in parentheses are *t* values.

**Table 3:** Endogeneity test.

Variables	Model 6	Model 7
Using or not using the Internet	0.223 ** (2.26)	0.188 * (1.92)
Gender	−0.179 ** (2.53)	0.119 * (1.71)
Age group	0.256 *** (4.34)	0.257 *** (4.40)
Education level	0.070 ** (2.12)	0.044 (1.36)
Marital status	0.422 *** (4.40)	0.410 *** (4.34)
Urban or rural area	0.165 ** (2.36)	0.147 ** (2.13)
Relative income level	0.207 *** (5.88)	0.199 *** (5.73)
Self-assessment of social status	0.390 *** (10.66)	0.336 *** (9.22)
Self-reported health	0.265 *** (9.74)	0.245 *** (9.11)
Bridging social capital: Popularity		0.167 *** (10.10)
Convergent social capital: Frequency of contact with children		0.193 *** (3.10)
Constant	4.152 *** (22.27)	2.968 *** (13.67)
Observations	3618	3618
*R*-squared	0.128	0.156
*F*	58.94	60.63

Note: *** *p* < 0.01, ** *p* < 0.05, * *p* < 0.1. The values in parentheses are *t* values.

**Table 4:** Analysis of the mediating effect of social capital.

M	X	Y = Subjective Well-Being				
		Sobel Test	Mediating Effect Coefficient	Direct Effect Coefficient	Overall Effect Coefficient	Proportion of Mediating Effect (%)
Bonding Social Capital	Internet use	0.061 ***	0.061 ***	0.266 ***	0.327 ***	18.69
Bridging Social Capital	Internet use	0.092 ***	0.092 ***	0.234 ***	0.327 ***	28.17

Note: M, mediation variable; X, independent variable; Y, dependent variable. *** *p* < 0.01.

**Table 5:** The moderating effect of marital status on the influence path of the intermediary mechanism.

Variables	Model 6	Model 7	Model 8	Model 9
	Explained Variable: Bonding Social Capital	Explained Variable: Bridging Social Capital	Explained Variable: Subjective Well-Being	Explained Variable: Subjective Well-Being
Use the Internet	0.249 *** (4.54)	−0.047 (−0.23)		
Convergent social capital: Frequency of contact with children			0.164 (1.25)	
Bridging social capital: Kinship				0.365 *** (10.97)
Gender	−0.071 *** (−4.17)	−0.234 *** (−3.76)	−0.087 (−1.38)	−0.005 (−0.09)
Age group	−0.107 *** (−7.37)	0.099 * (1.84)	0.303 *** (5.56)	0.231 *** (4.72)
Education level	0.044 *** (5.53)	0.063 ** (2.16)	0.075 *** (2.68)	0.057 ** (2.27)
Marital status	0.014 (0.27)	0.268 (1.40)	0.105 (0.51)	−0.308 (−1.11)
Urban or rural area	−0.007 (−0.39)	−0.041 (−0.66)	0.197 *** (3.18)	0.209 *** (3.72)
Relative income level	0.015 * (1.72)	0.037 (1.20)	0.195 *** (6.18)	0.185 *** (6.46)
Self-assessment of social status	0.018 ** (2.00)	0.401 *** (12.37)	0.351 *** (10.67)	0.180 *** (5.93)
Self-reported health	0.002 (0.30)	0.155 *** (6.43)	0.274 *** (11.21)	0.207 *** (9.32)
Using or not using the Internet × marital status	0.043 (0.76)	−0.293 (−1.39)		
Marital status × bonding social capital (unaccompanied as the reference group)				
Accompanied by a spouse			0.184 (1.29)	
Marital status × bridging social capital (unaccompanied as the reference group)				
Accompanied by a spouse				0.089 ** (2.47)
Constant	1.266 *** (27.57)	5.325 *** (31.57)	4.035 *** (16.69)	2.466 *** (8.81)
*n*	4463	4463	4463	4463
*R*-squared	0.070	0.072	0.128	0.286
*F*	33.44	34.69	65.63	178.12

Note: *** *p* < 0.01, ** *p* < 0.05, * *p* < 0.1; *t* values are in parentheses.

## Data Availability

http://www.isss.pku.edu.cn/cfps/download (accessed on 13 August 2022).
